# Deglutition Syncope: Two Case Reports Attributed to Vagal Hyperactivity

**DOI:** 10.1155/2017/2145678

**Published:** 2017-10-25

**Authors:** Sukhdeep Bhogal, Pooja Sethi, Yasir Taha, Muralidhar Papireddy, Akhilesh Mahajan, Syed Imran M. Zaidi, Vijay Ramu, Timir Paul

**Affiliations:** Department of Medicine, Division of Cardiology, East Tennessee State University, Johnson City, TN, USA

## Abstract

Deglutition syncope is a relatively rare cause of syncope that belongs to the category of neurally mediated reflex syncopal syndromes. The phenomenon is related to vagal reflex in context to deglutition causing atrioventricular block and acute reduction in cardiac output leading to dizziness or syncope. We present case series of two cases of deglutition syncope, of which first was managed medically and second with pacemaker implantation.

## 1. Introduction

Deglutition syncope (DS) is a relatively uncommon cause of syncope. Since the first reported case of swallow syncope in 1793 by Spens [1], there have been around 100 case reports published in the literature [2]. The usual presentation includes either presyncopal symptoms such as dizziness, lightheadedness, or overt syncope. It is more commonly seen in adult males [3]. The entity has been described to be associated with various esophageal disorders such as esophageal spasm, stricture, achalasia, hiatal hernia, and esophageal cancer [3–6]. However, the presence of functional and structural disorders of the esophagus is not mandatory for the diagnosis, as reported in many cases [7–9]. Besides this, almost 15% cases were associated with cardiac diseases such as myocardial infarction, [10] and in approximately 39% cases, the etiology or association remained unknown [11].

## 2. Case 1

**Figure 1:**
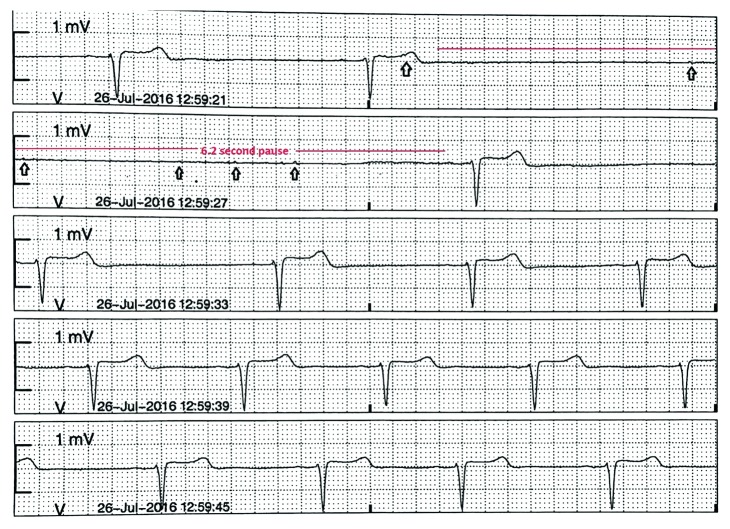
Telemetry strip showing sinus pause of 6.2 seconds, with atrial escape (marked P waves) with failed conductions to ventricles, likely representing high vagal tone during deglutition, followed by restoration of sinus rhythm (sinus arrhythmia).

A 68-year-old woman with a history of recently diagnosed glomerulonephritis, hypertension, and dyslipidemia was admitted with episodes of presyncope and dizziness. Her family history was unremarkable for arrhythmias or syncope. On admission, her heart rate (HR) was 54 beats per minute (bpm) and blood pressure was 166/94 mmHg with no evidence of orthostatic hypotension. Her home medications include amlodipine, losartan, and metoprolol tartrate (12.5 mg twice daily) for high blood pressure. The remainder of the physical examination including a comprehensive neurological examination was normal. Hematological and biochemical tests were unremarkable. A 12-lead electrocardiogram (ECG) showed sinus bradycardia with first degree atrioventricular block. Her echocardiogram and computed tomography of the head and neck were unremarkable as well. She was treated symptomatically with fluids. On the second day of admission, she had a syncopal spell with a 6.2 second pause, resulting in an atrial escape with failed conduction to ventricles, followed by restoration of sinus rhythm (sinus arrhythmia) which was noted on telemetry (Figure 1()) while swallowing her pills. The symptoms were reproduced by making her swallow cracker when her HR dropped from 66 bpm to 38 bpm, and she had another 3.8 second pause. Gastroenterology workup including a barium swallow showed small-sized hiatal hernia. She opted for medical management to be initiated with and was started on proton pump inhibitor with discontinuation of metoprolol. She had no more recurrence of symptoms while swallowing during her hospital stay. She was discharged with an event monitor and close cardiology follow-up. She continues to have syncopal episodes but reports incidence has decreased.

## 3. Case 2

**Figure 2:**
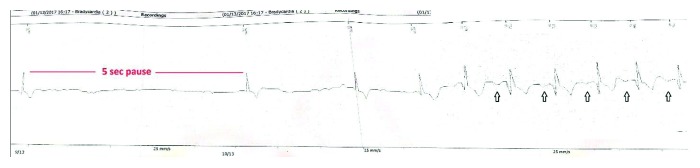
Loop recorder strip showing a 5-second pause followed by restoration of sinus rhythm (arrow showing marchable P waves).

A 59-year-old male presented to the hospital with recurrent episodes of lightheadedness while swallowing. He reports having several episodes of lightheadedness over the last thirteen years that have been occurring more frequently lately. He did not seek medical attention initially as episodes were occurring once or twice a year. However, over the past six months, he has been having these episodes at least once a month and had syncope during two of these episodes. He correlated these episodes with liquid diet only. His past medical history was insignificant, and he was not taking any medication. The patient was admitted for further evaluation. On admission, he was afebrile and had a blood pressure of 105/45 mmHg and a HR of 75 bpm. Physical examination including comprehensive neurological examination was unremarkable. Laboratory tests were normal. ECG showed normal sinus rhythm with occasional premature atrial complexes. Echocardiogram demonstrated normal EF of 55–60% with no evidence of diastolic dysfunction on Doppler filling pattern. Computed tomography of the head was unremarkable as well. Barium swallow did not show any organic disorder of the esophagus. Considering unexplained and infrequent symptoms, the patient underwent implantable loop recorder placement procedure. Prior to discharge, he had another episode of syncope with a 5-second pause (Figure 2()) noted on the loop recorder while drinking water. He was diagnosed with deglutition syncope and considered as a candidate for permanent pacemaker implantation. Following pacemaker implantation, he remains asymptomatic on six-month follow-up.

## 4. Discussion

The exact pathophysiology of DS remains unknown; several mechanisms have been postulated in the literature. In general, it is related to vagal reflex in context to deglutition causing temporary suppression of cardiovascular function. The mechanoreceptor in the esophagus is sensitive to stretch distension, and when activated, the signal is sent to the brainstem via the esophageal plexus [12]. The efferent signals are then mediated by right and left vagus nerves which innervate sinoatrial and atrioventricular nodes, respectively. Consequently, it leads to temporary bradyarrhythmias and sometimes a reduction in cardiac output causing hypotension via peripheral vasodilatation [11, 13]. Arrhythmias such as sinus bradycardia and atrial or ventricular asystole have been observed as well. Tachyarrhythmias such as atrial fibrillation have also been reported with DS [14, 15].

The diagnosis of DS involves obtaining careful history and recognition of syncopal or presyncopal symptoms with the specific type of meals. This can be further supported by provocative testing with food or other triggers [16]. Also, it is imperative to rule out any functional or organic esophageal disorder [11] with the barium swallow or endoscopy. After ruling out esophageal causes, cardiac workup should be considered which includes ECG, echocardiography, and an event monitor or inpatient telemetry to catch the symptomatic events [3], as done in our cases.

Various treatment options are available for DS. It is important to identify and avoid triggers before proceeding to medical management or pacemaker implantation. Certain foods such as carbonated drinks which are known to distend gastric lumen and triggering vagal response and resulting bradycardia should be avoided [2]. As there was no triggering factors and no functional or organic disorder of the esophagus (besides small hiatal hernia) present in our first case, medical management with discontinuation of beta blockers was considered. Discontinuation of medications that slow cardiac conduction such as beta blockers as well as which facilitate vasodepression such as angiotensin blockades or receptor inhibitors is considered as the mainstay of therapy [3] before proceeding with permanent pacemaker insertion. Furthermore, anticholinergic medications such as atropine can be used to prevent bradyarrhythmias secondary to vagal stimulation. Also, sympathomimetic agents like isoprenaline which was used to treat DS can be tried; however, their efficacy has not been well studied and use is also limited by their side effects [11]. Pacemaker implantation should be considered in patients who remain symptomatic even after avoiding triggering factors and withdrawing the potential culprit medications [11], which was considered in our second case resulting in resolution of syncopal events. As most of the workup was negative for any organic or functional disorder of the esophagus, high vagal tone during deglutition likely represents the mechanism of DS in both cases. Also, it may be worth to consider precautions such as avoidance of driving or operating heavy machinery while medical therapy is being tried or diagnosis is in question [17].

## 5. Conclusion

DS is a rare cause of syncope with limited data in the literature based on case reports, which can cause significant impairment in quality of life. Successful management involves careful history, avoidance of triggers if they are identified, removal of any potential atrioventricular nodal blocking medications, and the trial of sympathomimetic agents if well tolerated, and if all else fails, it needs pacemaker implantation.
